# Dataset on rbcL-based intra-specific diversity and population structure of *Parkia biglobosa* (Jacq.) in Nigeria

**DOI:** 10.1016/j.dib.2024.110146

**Published:** 2024-02-05

**Authors:** Conrad A. Omonhinmin, Nchedo S. Taiwo, Paul B. Okonkwor, Israel M. Ajayi, Paul Akinniyi Akinduti, Oluwadurotimi S. Aworunse, Ibukun Ajiboye, Olugbenga S. Taiwo, Bosede Temitope Adekeye, Olubukola Oziegbe, Adetutu O. Bello, Eze Frank Ahuekwe, Joshua Oyekanmi, Olanrewaju Olufowobi, Margaret Ikhiwili Oniha, Oyewumi Oshamika, Samuel A. Ejoh, Adeyemi G. Adewale, Olayemi O. Akinnola, Solomon U. Oranusi, Jacob O. Popoola

**Affiliations:** a Department of Biological Sciences/Biotechnology Cluster, Covenant University, Ota, Ogun State, Nigeria; b Pure and Applied Biology Programme, College of Agriculture, Engineering and Science, Bowen University, Iwo, Nigeria; c Inqaba Biotec West Africa Limited, Ibadan, Oyo State, Nigeria; d Department of Civil Engineering, Covenant University, Ota, Ogun State, Nigeria

**Keywords:** African locust bean, Breeding, Conservation, Genetic diversity, Phylogenetic, rbcL, *Parkia biglobosa*

## Abstract

African locust bean (*Parkia biglobosa*) is a multipurpose leguminous tree species of nutritional and pharmacological value. The plant is widely distributed in Africa and across Nigeria's major agroecological areas (AEAs). Amidst declining cultivation and production, *P. biglobosa* is genetically threatened in its natural habitats due to overexploitation, deforestation, wildfires and lack of improved tree management practices. Consequently, concerted research efforts directed towards germplasm collection and assessment of genetic relationships are imperative for conserving its genetic resources, sustainable management and selecting promising landraces for breeding programmes. The dataset presents rbcL intraspecific genetic diversity and population structure of 62 *P. biglobosa* landraces in Nigeria. A relatively high level of diversity and a low degree of nucleotide variability was observed among the landraces. Relatively high values of 642 total allele sites, 601 polymorphic sites, 504 parsimony information sites, 883 total number mutations, 9 haplotypes and 0.55 gene diversity were recorded for the sequence dataset. Low values of 0.35 nucleotide diversity and 5 InDels events were also recorded for the dataset. The gene flow in this dataset demonstrated an extensive exchange of genes between the three populations of *P. biglobosa,* which influenced the level of genetic differentiation (Gst) between the populations. Significantly low Gst (-0.01) was recorded between the Guinea and Sudan savannah populations, a moderate value (0.03) was recorded between the Sudan savannah and Rainforest populations and a higher Gst value (0.05) was recorded between the Guinea and Rainforest populations. The dataset highlights potential evolutionary dynamics that might influence variations relevant to the breeding and conservation of *P. biglobosa* in Nigeria and across its range in West and Central Africa.

Specifications Table

**Table:** 

Subject	Biological Science
Specific subject area	Plant Science, Genetic diversity, Phylogeny, and Evolution, Bioinformatics
Type of data	Tables, Figure
How the data were acquired	Amplification of the ribulose-1,5-bisphosphate carboxylase/oxygenase large subunit (rbcL) gene, partial cds; chloroplast through PCR and Sanger Sequencing of DNA. Data were analysed using BioEdit, DnaSP v6.12.03, and MEGAX.
Data format	Raw Analysed
Description of data collection	A total of sixty-two (62) landraces of African locust bean (*P. biglobosa*) were collected from the major agroecological areas (AEAs) (Rainforest, Guinea Savannah and Sudan savannah) of Nigeria. Partial rbcL sequences were employed to evaluate intraspecific genetic diversity and population structure among the landraces. All 62 sequences were aligned using ClustalX2.1. Nucleotide sequence statistics were performed using MEGAX and CodonW. The genetic diversity and population structure, gene flow, and genetic differentiation among different populations of *P. biglobosa* were performed using DnaSP v6.12.03. The total number of sites, invariable sites, parsimony information sites, the total number of mutations (Eta), the number of haplotypes, gene diversity, the variance of haplotypes, nucleotide diversity and the total number of insertion and deletions (InDels) and the total number of
	InDels events were evaluated. Haplotype-based statistics, the average proportion of nucleotide difference between populations, the genetic differentiation index based on the frequency of haplotypes, the average number of nucleotide substitutions per site between populations, and the net nucleotide substitutions per site between populations were also estimated. Data were also recorded for codon and its indices per sequence accession of *P. biglobosa*.
Data source location	The collection areas and distribution map of the *Parkia biglobosa* are summarised in Table 1 and Figure 1.
Data accessibility	There are two PopSet Repository name: I (PopSet) PopSet 1 Accession number: 2280517159 [1] Direct URL to data: https://www.ncbi.nlm.nih.gov/popset/?term=2280517159 PopSet 2 Accession number: 2304762196 [2] Direct URL to data: https://www.ncbi.nlm.nih.gov/popset/?term=2304762196

## 1 Value of the Data

• Based on sequences data, the species range and the three major endemic agro-ecological areas are recognised for Nigeria and by extension, the West-Central Africa regions. These include; the Guinea savannah, the Sudan savannah, and the Rainforest.
• The area(s) of greater gene diversity for the species across the agro-ecological regions in Nigeria are highlighted and is in the increasing order of Guinea savannah (0.43), Sudan savannah (0.62) and the Rainforest (0.73).
• The Sudan savannah population is observing a greater degree of genetic variation, probably due to anthropogenic and climate change pressures and displayed higher number of mutations, total number of segregating sites, the haplotype number and average number of nucleotide differences between sequences.
• The genetic information provided by the dataset offers genome-based species recovery, conservation and genetic improvement strategies like Genome-wide association studies **(**GWAS), haplotype-assisted genomic selection, and haplotype-based breeding to augment the natural hybridisation of the species.
• The sequence data on *P. biglobosa* are integral for re-cultivation, genetic characterisation, conservation and species improvement by breeders and scientists.

## 2 Objectives

The objectives of the dataset are to determine intraspecific genetic diversity, population structure, and gene flow among three agro-ecological populations of African locust beans (*Parkia biglobosa*) in Nigeria.

## 3 Data Description

**Fig. 1:**
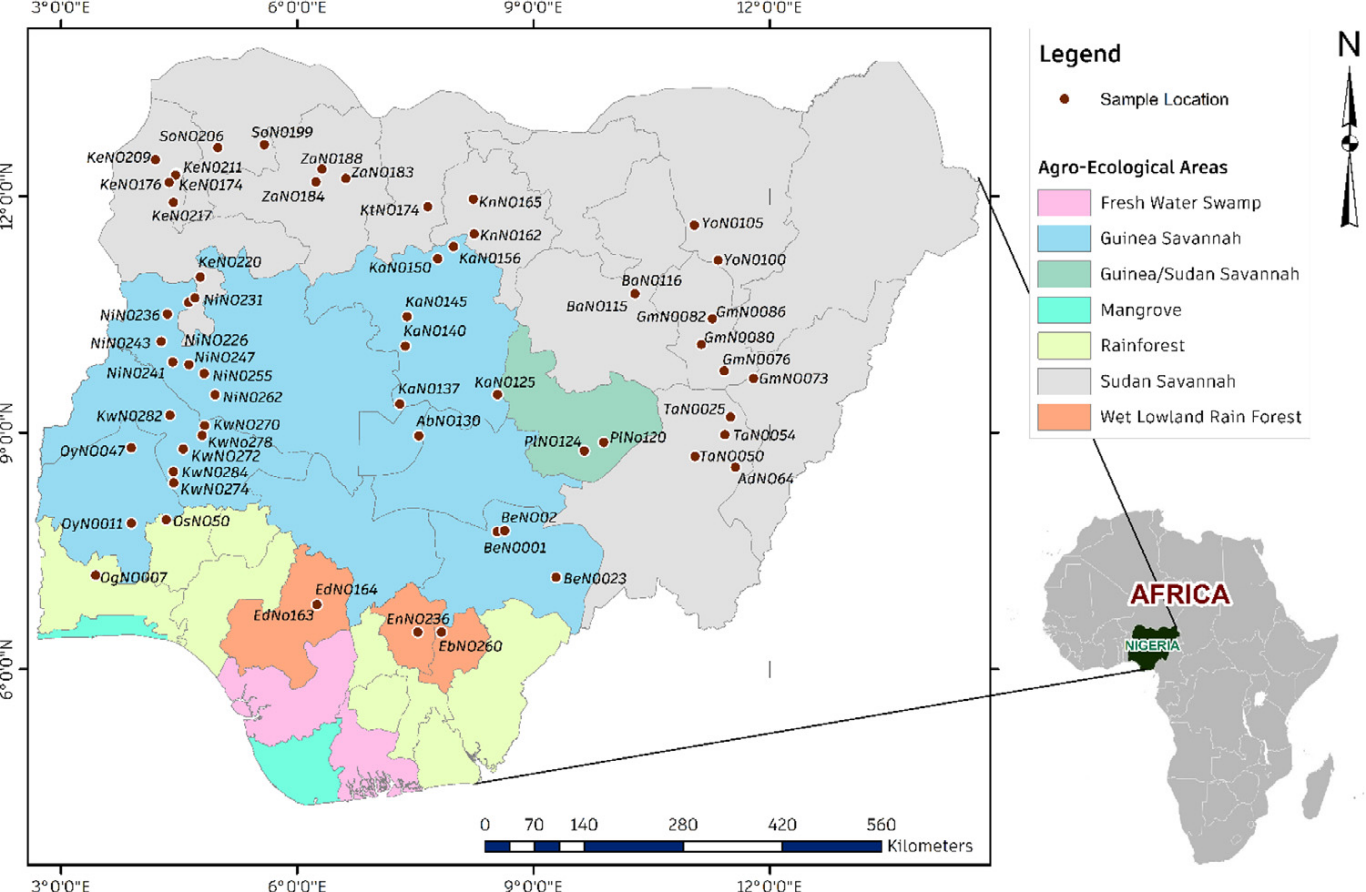
Distribution pattern and the Agro-ecological categories of the collection sites for *Parkia biglobosa* landraces (Pattern shows *P. biglobosa* is distributed outside the very wet areas).

The data presents genetic intraspecific diversity and population structure of *Parkia biglobosa* across various agro-ecological areas in Nigeria and West-Central Africa. Sixty-two landraces of three populations representing the major agro-ecological areas - AEAs (Rainforest, Guinea Savannah, Sudan savannah). PCR amplification and Sanger Sequencing using the Ribulose-1,5-bisphosphate carboxylase/oxygenase large subunit (rbcL) gene were performed on the samples. Table 1 describes the collection areas and AEAs of *P. biglobosa* landraces; Fig. 1 shows the landraces distribution in Nigeria across the AEAs. Table 2 describes nucleotide sequences statistics; sequence length (bp), the weight of single and double DNA strands, the frequencies of nucleotide bases (A, T, G, C; C + G, and A + T) and the total number of codons per sequence accession. Table 3 presents the genetic diversity parameters such as the total number of sites, invariable sites, parsimony information sites, the total number of mutations (Eta), number of haplotypes, gene diversity, the variance of haplotypes, nucleotide diversity, the total number of insertion and deletions (InDels) and the total number of InDels events. Table 4 records the multidomain analysis and population structure of the three populations of the sixty-two landraces, whereas Table 5 presents the gene flow and genetic differentiations among the three *P. biglobosa* populations. Table 6 shows the codon usage and amino residues of the *P. biglobosa* sequences analysed for the 62 accessions. Table 7 records the codon usage indices per sequence accession of the 62 *P. biglobosa* landraces.

**Table 1:** Collection location, Sample ID, Agro-ecological areas, and Geodetic coordinate system for the *Parkia biglobosa accessions* landraces studied.

SN	Original Sample ID§	Location	State	(AEAs)	Latitude (°N)	Longitude (°E)	Altitude (m)
1	AbNO130	Abaji	Abuja	Guinea Savannah	6.945068	8.474675	137
2	AdNO64	Jimeta	Adamawa	Sudan Savannah	9.33333	12.5	135
3	BaNO115	Gurzumo	Bauchi	Sudan Savannah	10.766	10.288	510
4	BaN0116	Soro	Bauchi	Sudan Savannah	10.7773	10.2976	504
5	BeN0001	North bank	Benue	Guinea Savannah	7.753380	8.547500	173
6	BeNO02	Katsina-Ala	Benue	Guinea Savannah	7.1658	9.2841	144
7	BeN0023	Katsina-Ala	Benue	Guinea Savannah	7.167	9.287	145
8	EbNO260	Ishi Elu	Ebonyi	Rainforest	6.3907	7.8286	51
9	EdNo163	Ehanlen-Ewu	Edo	Rainforest	6.77973	6.2530	381
10	EdNO164	Ehanlen-Ewu	Edo	Rainforest	6.82	6.25	379
11	EnNO236	Thinkers corner	Enugu	Rainforest	6.4742	7.5518	142
12	GmNO073	Lafia-Lamurde	Gombe	Sudan Savannah	9.69	11.791	651
13	GmN0076	Ture-Balam	Gombe	Sudan Savannah	9.787	11.42	457
14	GmN0080	Kashere	Gombe	Sudan Savannah	10.124	11.131	368
15	GmN0082	Doho	Gombe	Sudan Savannah	10.447	11.267	439
16	GmN0086	Azume	Gombe	Sudan Savannah	10.451	11.27	335
17	KaN0125	Aboro Village	Kaduna	Guinea Savannah	8.542	9.39617	634
18	KaN0137	Igwa	Kaduna	Guinea Savannah	9.3887	7.2820	545
19	KaN0140	Karasami	Kaduna	Guinea Savannah	10.1762	7.2911	635
20	KaN0145	Defence Academy	Kaduna	Guinea Savannah	10.6155	7.3663	732
21	KaN0150	Rafin Gwaza	Kaduna	Guinea Savannah	11.2020	7.7974	742
22	KaN0156	Kwabo	Kaduna	Guinea Savannah	11.363	7.983	751
23	KeNo174	Birni	Kebbi	Sudan Savannah	12.466	4.199	281
24	KeNO176	Ailero	Kebbi	Sudan Savannah	12.289	4.464	258
25	KeNO209	Besse	Kebbi	Sudan Savannah	12.271	4.453	243
26	KeN0211	Maiyama	Kebbi	Sudan Savannah	12.178	4.372	245
27	KeN0217	Dada Alelu	Kebbi	Sudan Savannah	11.924	4.427	238
28	KeNO220	Kebbi	Kebbi	Sudan Savannah	10.978	4.768	236
29	KnN0162	Gwarmai	Kano	Sudan Savannah	11.524	8.246	575
30	KnNO165	Rimin Gado	Kano	Sudan Savannah	11.966	8.235	520
31	KwNO270	Pako	Kwara	Guinea Savannah	9.091	4.825	345
32	KwN0272	Jebba	Kwara	Guinea Savannah	8.966	4.792	98
33	KwN0274	Jebba	Kwara	Guinea Savannah	8.966	4.792	98
34	KwNo278	Pakunmo	Kwara	Guinea Savannah	8.795	4.552	99
35	KwN0282	Ballah/Otte	Kwara	Guinea Savannah	8.507	4.427	298
36	KwN0284	Ballah/Otte	Kwara	Guinea Savannah	8.366	4.431	307
37	KtNO174	Yarmama	Katsina	Sudan Savannah	11.871	7.657	613
38	NiNO132	Suteku	Niger	Guinea Savannah	10.656	4.621	547
39	NiN0226	Jinjima	Niger	Guinea Savannah	10.7105	4.7080	139
40	NiNO231	Zugu	Niger	Guinea Savannah	10.508	4.351	219
41	NiN0236	Shagunui	Niger	Guinea Savannah	10.16	4.273	311
42	NiN0241	Wawa	Niger	Guinea Savannah	9.899	4.419	202
43	NiN0243	Nasarawa, Lapai	Niger	Guinea Savannah	9.042490	6.570960	142
44	NiNO247	Ibbi	Niger	Guinea Savannah	9.662440	4.906710	144
45	NiN0255	Ibbi	Niger	Guinea Savannah	9.662440	4.906710	144
46	NiN0262	Tashabu	Niger	Guinea Savannah	9.224	4.384	154
47	OgNO007	Osiele	Ogun	Rainforest	7.1608	3.3483	158
48	OsNO50	Ejigbo	Osun	Rainforest	7.9045	4.3052	426
49	OyN0011	Iseyin road	Oyo	Guinea Savannah	7.8537	3.8932	321
50	OyNO047	Ogbomoso	Oyo	Guinea Savannah	8.81	3.89	347
51	PlNo120	Kumbur	Plateau	Guinea Savannah	8.878927	9.890224	197
52	PlNO124	Shendam	Plateau	Guinea Savannah	8.771	9.644	218
53	SoN0199	Lamba-Tureta	Sokoto	Sudan Savannah	12.658	5.581	311
54	SoNO206	Shagari	Sokoto	Sudan Savannah	12.621	4.99	324
55	TaN0025	Yoina, Wukari	Taraba	Sudan Savannah	7.768	7.903	191
56	TaNO050	Jauro Kurungu	Taraba	Sudan Savannah	8.7268	11.0489	174
57	TaN0054	Kukaliri	Taraba	Sudan Savannah	8.013748	10.800537	185
58	YoN0100	Gadaka	Yobe	Sudan Savannah	11.3322	11.2297	366
59	YoN0105	Potiskum	Yobe	Sudan Savannah	11.7072	11.0825	475
60	ZaNO183	Furfuri Kwaikwai	Zamfara	Sudan Savannah	12.18238	6.5056	447
61	ZaNO184	Maradun	Zamfara	Sudan Savannah	12.56667	6.24444	465
62	ZaN0188	Kadauri	Zamfara	Sudan Savannah	12.347	6.326	449

§ All samples were identified by Botanists – C. A. Omonhinmin and J. O. Popoola and assigned sample identity numbers (Original Sample ID).

**Table 2:** Nucleotide sequence statistics of the 62- *Parkia biglobosa* accessions studied.

Original Sample ID	GenBank Accession No.	SL (bp)	WTss (kDa)	WTds (kDa)	Fr (A)	Fr (C)	Fr (G)	Fr (T)	C + G	A + T	NCo
AbNO130	ON864386.1	663	205.03	409.66	0.267	0.213	0.232	0.288	0.445	0.555	221
AdNO64	ON864387.1	663	205.03	409.66	0.267	0.213	0.232	0.288	0.445	0.555	221
BaNO115	ON864388.1	668	206.59	412.75	0.266	0.213	0.234	0.287	0.446	0.554	222
BaN0116	OP178899.1	642	198.46	396.69	0.266	0.212	0.227	0.294	0.439	0.561	214
BeN0001	OP178900.1	642	198.46	396.69	0.266	0.212	0.227	0.294	0.439	0.561	214
BeNO02	ON864389.1	662	204.74	409.05	0.266	0.213	0.234	0.287	0.447	0.553	220
BeN0023	OP178902.1	642	198.46	396.69	0.266	0.212	0.227	0.294	0.439	0.561	214
EbNO260	ON864390.1	646	199.75	399.16	0.266	0.214	0.232	0.286	0.446	0.553	214
EdNo163	OP178904.1	642	198.46	396.69	0.266	0.212	0.227	0.294	0.439	0.561	214
EdNO164	ON864391.1	668	206.55	412.75	0.266	0.214	0.232	0.287	0.446	0.554	222
EnNO236	ON864392.1	662	204.74	409.05	0.266	0.213	0.234	0.287	0.447	0.553	222
GmNO073	OP178907.1	642	198.46	396.69	0.266	0.212	0.227	0.294	0.439	0.561	214
GmN0076	OP178908.1	642	198.46	396.69	0.266	0.212	0.227	0.294	0.439	0.561	214
GmN0080	OP178909.1	642	198.46	396.69	0.266	0.212	0.227	0.294	0.439	0.561	214
GmN0082	OP178910.1	642	198.50	396.69	0.266	0.21	0.229	0.294	0.439	0.561	214
GmN0086	OP178911.1	642	198.46	396.69	0.266	0.212	0.227	0.294	0.439	0.561	214
KaN0125	OP178912.1	642	198.46	396.69	0.266	0.212	0.227	0.294	0.439	0.561	214
KaN0137	OP178913.1	642	198.46	396.69	0.266	0.212	0.227	0.294	0.439	0.561	214
KaN0140	OP178914.1	642	198.46	396.69	0.266	0.212	0.227	0.294	0.439	0.561	214
KaN0145	OP178915.1	642	198.46	396.69	0.266	0.212	0.227	0.294	0.439	0.561	214
KaN0150	OP178916.1	642	198.46	396.69	0.266	0.212	0.227	0.294	0.439	0.561	214
KaN0156	OP178917.1	642	198.46	396.69	0.266	0.212	0.227	0.294	0.439	0.561	214
KeNo174	OP178920.1	642	198.46	396.69	0.266	0.212	0.227	0.294	0.439	0.561	214
KeNO176	OP178921.1	642	198.46	396.69	0.266	0.212	0.227	0.294	0.439	0.561	214
KeNO209	ON864394.1	668	206.60	412.75	0.268	0.213	0.234	0.286	0.446	0.554	222
KeN0211	OP178923.1	642	198.46	396.69	0.266	0.212	0.227	0.294	0.439	0.561	214
KeN0217	OP178924.1	642	198.46	396.69	0.266	0.212	0.227	0.294	0.439	0.561	214
KeNO220	ON864395.1	661	204.41	408.43	0.266	0.213	0.233	0.287	0.446	0.554	220
KnN0162	OP178918.1	642	198.46	396.69	0.266	0.212	0.227	0.294	0.439	0.561	214
KnNO165	ON864396.1	668	206.55	412.75	0.266	0.214	0.232	0.287	0.446	0.554	222
KwNO270	ON864397.1	668	206.59	412.75	0.266	0.213	0.234	0.287	0.446	0.554	222
KwN0272	OP178927.1	642	198.46	396.69	0.266	0.212	0.227	0.294	0.439	0.561	214
KwN0274	OP178928.1	642	198.46	396.69	0.266	0.212	0.227	0.294	0.439	0.561	214
KwNo278	ON864398.1	668	206.59	412.75	0.266	0.213	0.234	0.287	0.446	0.554	222
KwN0282	OP178930.1	642	198.46	396.69	0.266	0.212	0.227	0.294	0.439	0.561	214
KwN0284	OP178931.1	642	198.46	396.69	0.266	0.212	0.227	0.294	0.439	0.561	214
KtNO174	ON864399.1	668	206.59	412.75	0.266	0.213	0.234	0.287	0.446	0.554	222
NiNO132	OP178932.1	642	198.46	396.69	0.266	0.212	0.227	0.294	0.439	0.561	214
NiN0226	OP178933.1	642	198.46	396.69	0.266	0.212	0.227	0.294	0.439	0.561	214
NiNO231	ON864400.1	668	206.59	412.75	0.266	0.213	0.234	0.287	0.446	0.554	222
NiN0236	OP178935.1	642	198.46	396.69	0.266	0.212	0.227	0.294	0.439	0.561	214
NiN0241	OP178936.1	642	198.46	396.69	0.266	0.212	0.227	0.294	0.439	0.561	214
NiN0243	OP178937.1	642	198.46	396.69	0.266	0.212	0.227	0.294	0.439	0.561	214
NiNO247	ON864401.1	668	206.59	412.75	0.266	0.213	0.234	0.287	0.446	0.554	222
NiN0255	OP178939.1	642	198.46	396.69	0.266	0.212	0.227	0.294	0.439	0.561	214
NiN0262	OP178940.1	642	198.46	396.69	0.266	0.212	0.227	0.294	0.439	0.561	214
OgNO007	OP178941.1	642	198.46	396.69	0.266	0.212	0.227	0.294	0.439	0.561	214
OsNO50	ON864402.1	668	206.59	412.75	0.266	0.213	0.234	0.287	0.446	0.554	222
OyN0011	OP178943.1	642	198.40	396.69	0.259	0.213	0.227	0.301	0.441	0.559	214
OyNO047	OP178944.1	642	198.46	396.69	0.266	0.212	0.227	0.294	0.439	0.561	214
PlNo120	OP178945.1	642	198.46	396.69	0.266	0.212	0.227	0.294	0.439	0.561	214
PlNO124	ON864403.1	668	206.59	412.75	0.266	0.213	0.234	0.287	0.446	0.554	222
SoN0199	OP178947.1	642	198.46	396.69	0.266	0.212	0.227	0.294	0.439	0.561	214
SoNO206	ON864404.1	662	204.75	409.05	0.266	0.213	0.234	0.285	0.447	0.551	219
TaN0025	OP178949.1	642	198.41	396.68	0.273	0.213	0.223	0.291	0.436	0.564	214
TaNO050	ON864405.1	668	206.55	412.75	0.274	0.214	0.229	0.283	0.443	0.557	222
TaN0054	OP178951.1	642	198.46	396.69	0.266	0.212	0.227	0.294	0.439	0.561	214
YoN0100	OP178952.1	642	198.46	396.69	0.266	0.212	0.227	0.294	0.439	0.561	214
YoN0105	OP178953.1	642	198.46	396.69	0.266	0.212	0.227	0.294	0.439	0.561	214
ZaNO183	ON864406.1	662	204.77	409.05	0.266	0.213	0.236	0.285	0.449	0.551	220
ZaNO184	OP178955.1	642	198.46	396.69	0.266	0.212	0.227	0.294	0.439	0.561	214
ZaN0188	OP178956.1	642	198.46	396.69	0.266	0.212	0.227	0.294	0.439	0.561	214

**SL** = Sequence Length, **WTss** = Weight of single strand, **WTds** = Weight of double-strand, **Fr (A)** = Frequency of Adenine, **Fr (C) =** Frequency of Cytosine.

**Fr (G)** = Frequency of Guanine, **Fr (T)** = Frequency of Thiamine, **C + G** = Cytosine + Guanine contents, **A+T** = Adenine + Thiamine contents.

**NCo** = Number of Codons.

**Table 3:** Genetic diversity, insertion and deletion (InDels) polymorphisms among 62 sequences of *P. biglobosa* studied.

Index	Value
No. of nucleotides	62
Total number of sites (excluding sites with gaps / missing data	642
Invariable (monomorphic) sites	41
No. of polymorphic (segregating sites)	601
Parsimony informative sites	504
Total number of mutations (Eta)	883
No. of haplotypes (h)	9
Haplotype (gene) diversity (Hd)	0.545
The variance of Haplotype diversity + SD	0.0034 ± 0.058
Nucleotide diversity (Pi)	0.35
The average number of nucleotide differences (k)	221.85
Total number of InDel sites	26
Total number of (InDel and non-InDel) sites analysed	642
Total number of InDels events	5

**Table 4:** Multidomain analysis and population structure of the 62 *Parkia biglobosa* accessions.

AEAs	Populations	N	S	Eta	K	ThetaNu	Pi (π)	Hap	Hd	VarHD	TajimaD*	FuLiD*	FuLif*	G+Cn	G+Ctot
Guinea	Pop 1	29	488	495	184.95	126.04	0.293	3	0.43	0.008	1.83	−4.57	−4.31	0.45	0.45
Sudan	Pop 2	27	593	856	235.03	222.08	0.37	7	0.62	0.008	0.08	−4.37	−4.15	0.44	0.44
Rainforest	Pop 3	6	509	557	277.6	243.94	0.43	3	0.73	0.02	0.90	−1.24	−1.24	0.44	0.44
Total	3	62													

**AEAs:** Agro-Ecological Areas; **Guinea:** Guinea Savannah; **Sudan**: Sudan Savannah; **N**: Number of sequences per population; **S**: Total number of Polymorphic sites; **Eta**: Total number of mutations; **K**: Average number of nucleotide differences between lines; **ThetaNu**: mutation rate per population/sequences; **Pi (π)**: Nucleotide diversity; **Hap**: Haplotype number; **Hd**: Haplotype diversity; **VarHd**: Haplotype diversity variance; D, D*, F, and F* statistics test various predictions of the neutral theory of molecular evolution [[1], [2]] and their significance.

*p < 0.1, G+C, G+C content; tot, -total.

**Table 5:** Gene flow and genetic differentiation among the 3 populations of the 62 *Parkia biglobosa* accessions studied.

Population 1	Population 2	Hs	Ks	Kxy	Gst	DeltaSt	GammaSt	Nst	Fst	Dxy	Da
Population_1	Population_2	0.52	209.1	205.5	−0.01	0	0.01	−0.06	−0.02	0.32	−0.01
Population_1	Population_3	0.47	200.83	289.31	0.05	0.04	0.11	−1.18	0.2	0.45	0.09
Population_2	Population_3	0.63	242.77	287.22	0.03	0.03	0.07	0.22	0.11	0.45	0.05

**Hs** = haplotype-based statistics; **Ks** = statistics based on nucleotide sequences, **Kxy** = average proportion of nucleotide difference between populations; **Gst** = genetic differentiation index based on the frequency of haplotypes; **GammaS**t = genetic differentiation coefficient; **Fst** = genetic differentiation; **Dxy** = average number of nucleotide substitutions per site between populations; **Da** = net nucleotide substitutions per site between populations.

**Table 6:** Codon usage for the 62 *Parkia biglobosa* accessions landraces.

Codon		Freq	RSCU	Codon		Freq	RSCU	Codon		Freq	RSCU	Codon		Freq	RSCU
Phe	UUU	7	1.27	Ser	UCU	5	3	Tyr	UAU	7	1.17	Cys	UGU	3	1.5
	UUC	4	0.73		UCC	3	1.8		UAC	5	0.83		UGC	1	0.5
Leu	UUA	3	0.95		UCA	0	0	TER	UAA	0	0	TER	UGA	0	0
	UUG	6	1.89		UCG	0	0		UAG	0	0	Trp	UGG	3	1
	CUU	5	1.58	Pro	CCU	7	1.87	His	CAU	1	0.67	Arg	CGU	6	2.77
	CUC	1	0.32		CCC	3	0.8		CAC	2	1.33		CGC	1	0.46
	CUA	2	0.63		CCA	3	0.8	Gln	CAA	5	1.67		CGA	3	1.38
	CUG	2	0.63		CCG	2	0.53		CAG	1	0.33		CGG	0	0
Ile	AUU	3	1.12	Thr	ACU	10	2.5	Asn	AAU	5	1.43	Ser	AGU	2	1.2
	AUC	5	1.88		ACC	3	0.75		AAC	2	0.57		AGC	0	0
	AUA	0	0		ACA	3	0.75	Lys	AAA	10	1.67	Arg	AGA	3	1.38
Met	AUG	2	1		ACG	0	0		AAG	2	0.33		AGG	0	0
Val	GUU	5	1.54	Ala	GCU	7	1.47	Asp	GAU	9	1.5	Gly	GGU	9	1.8
	GUC	0	0		GCC	4	0.84		GAC	3	0.5		GGC	2	0.4
	GUA	5	1.54		GCA	5	1.05	Glu	GAA	12	1.5		GGA	5	1
	GUG	3	0.92		GCG	3	0.63		GAG	4	0.5		GGG	4	0.8

Average# codons=216.

**Freq** = Frequency. All frequencies are averages over all taxa. **RSCU** - Relative synonymous codon usage.

**Table 7:** Codon usage parameters per accession for the 62 *P. biglobosa* accessions.

Accessions	T3s	C3s	A3s	G3s	CAI	CBI	Fop	Nc	GC3s	GC	L_sym	L_aa	Gravy	Aromo
ON864386.1	0.5	0.2143	0.3533	0.1698	0.274	0.095	0.477	49.36	0.306	0.445	216	221	−0.390	0.118
ON864387.1	0.5	0.2143	0.3533	0.1698	0.274	0.095	0.477	49.36	0.306	0.445	216	221	−0.390	0.118
ON864388.1	0.5027	0.2131	0.3512	0.1688	0.274	0.097	0.479	49.57	0.304	0.446	217	222	−0.413	0.117
OP178899.1	0.5114	0.2102	0.35	0.1699	0.27	0.082	0.469	47.77	0.301	0.439	209	214	−0.432	0.126
OP178900.1	0.5114	0.2102	0.35	0.1699	0.27	0.082	0.469	47.77	0.301	0.439	209	214	−0.432	0.126
ON864389.1	0.5028	0.2155	0.3494	0.1709	0.274	0.097	0.479	49.44	0.307	0.445	215	220	−0.411	0.118
OP178902.1	0.5114	0.2102	0.35	0.1699	0.27	0.082	0.469	47.77	0.301	0.439	209	214	−0.432	0.126
ON864390.1	0.4629	0.2229	0.3152	0.2338	0.252	0.109	0.476	54.03	0.361	0.447	208	214	−0.283	0.103
OP178904.1	0.5114	0.2102	0.35	0.1699	0.27	0.082	0.469	47.77	0.301	0.439	209	214	−0.432	0.126
ON864391.1	0.4973	0.2131	0.3571	0.1688	0.273	0.092	0.475	49.45	0.304	0.446	217	222	−0.396	0.117
ON864392.1	0.5028	0.2155	0.3494	0.1709	0.274	0.097	0.479	49.44	0.307	0.445	215	220	−0.411	0.118
OP178907.1	0.5114	0.2102	0.35	0.1699	0.27	0.082	0.469	47.77	0.301	0.439	209	214	−0.432	0.126
OP178908.1	0.5114	0.2102	0.35	0.1699	0.27	0.082	0.469	47.77	0.301	0.439	209	214	−0.432	0.126
OP178909.1	0.5114	0.2102	0.35	0.1699	0.27	0.082	0.469	47.77	0.301	0.439	209	214	−0.432	0.126
OP178910.1	0.5114	0.2102	0.35	0.1699	0.274	0.09	0.474	48.01	0.301	0.439	209	214	−0.445	0.126
OP178911.1	0.5114	0.2102	0.35	0.1699	0.27	0.082	0.469	47.77	0.301	0.439	209	214	−0.432	0.126
OP178912.1	0.5114	0.2102	0.35	0.1699	0.27	0.082	0.469	47.77	0.301	0.439	209	214	−0.432	0.126
OP178913.1	0.5114	0.2102	0.35	0.1699	0.27	0.082	0.469	47.77	0.301	0.439	209	214	−0.432	0.126
OP178914.1	0.5114	0.2102	0.35	0.1699	0.27	0.082	0.469	47.77	0.301	0.439	209	214	−0.432	0.126
OP178915.1	0.5114	0.2102	0.35	0.1699	0.27	0.082	0.469	47.77	0.301	0.439	209	214	−0.432	0.126
OP178916.1	0.5114	0.2102	0.35	0.1699	0.27	0.082	0.469	47.77	0.301	0.439	209	214	−0.432	0.126
OP178917.1	0.5114	0.2102	0.35	0.1699	0.27	0.082	0.469	47.77	0.301	0.439	209	214	−0.432	0.126
OP178920.1	0.5114	0.2102	0.35	0.1699	0.27	0.082	0.469	47.77	0.301	0.439	209	214	−0.432	0.126
OP178921.1	0.5114	0.2102	0.35	0.1699	0.27	0.082	0.469	47.77	0.301	0.439	209	214	−0.432	0.126
ON864394.1	0.4973	0.2131	0.3571	0.1688	0.268	0.089	0.475	49.79	0.304	0.446	217	222	−0.413	0.117
OP178923.1	0.5114	0.2102	0.35	0.1699	0.27	0.082	0.469	47.77	0.301	0.439	209	214	−0.432	0.126
OP178924.1	0.5114	0.2102	0.35	0.1699	0.27	0.082	0.469	47.77	0.301	0.439	209	214	−0.432	0.126
ON864395.1	0.5028	0.2155	0.3494	0.1709	0.274	0.097	0.479	49.44	0.307	0.445	215	220	−0.411	0.118
OP178918.1	0.5114	0.2102	0.35	0.1699	0.27	0.082	0.469	47.77	0.301	0.439	209	214	−0.432	0.126
ON864396.1	0.4973	0.2131	0.3571	0.1688	0.273	0.092	0.475	49.45	0.304	0.446	217	222	−0.396	0.117
ON864397.1	0.5027	0.2131	0.3512	0.1688	0.274	0.097	0.479	49.57	0.304	0.446	217	222	−0.413	0.117
OP178927.1	0.5114	0.2102	0.35	0.1699	0.27	0.082	0.469	47.77	0.301	0.439	209	214	−0.432	0.126
OP178928.1	0.5114	0.2102	0.35	0.1699	0.27	0.082	0.469	47.77	0.301	0.439	209	214	−0.432	0.126
ON864398.1	0.5027	0.2131	0.3512	0.1688	0.274	0.097	0.479	49.57	0.304	0.446	217	222	−0.413	0.117
OP178930.1	0.5114	0.2102	0.35	0.1699	0.27	0.082	0.469	47.77	0.301	0.439	209	214	−0.432	0.126
OP178931.1	0.5114	0.2102	0.35	0.1699	0.27	0.082	0.469	47.77	0.301	0.439	209	214	−0.432	0.126
ON864399.1	0.5027	0.2131	0.3512	0.1688	0.274	0.097	0.479	49.57	0.304	0.446	217	222	−0.413	0.117
OP178932.1	0.5114	0.2102	0.35	0.1699	0.27	0.082	0.469	47.77	0.301	0.439	209	214	−0.432	0.126
OP178933.1	0.5114	0.2102	0.35	0.1699	0.27	0.082	0.469	47.77	0.301	0.439	209	214	−0.432	0.126
ON864400.1	0.5027	0.2131	0.3512	0.1688	0.274	0.097	0.479	49.57	0.304	0.446	217	222	−0.413	0.117
OP178935.1	0.5114	0.2102	0.35	0.1699	0.27	0.082	0.469	47.77	0.301	0.439	209	214	−0.432	0.126
OP178936.1	0.5114	0.2102	0.35	0.1699	0.27	0.082	0.469	47.77	0.301	0.439	209	214	−0.432	0.126
OP178937.1	0.5114	0.2102	0.35	0.1699	0.27	0.082	0.469	47.77	0.301	0.439	209	214	−0.432	0.126
ON864401.1	0.5027	0.2131	0.3512	0.1688	0.274	0.097	0.479	49.57	0.304	0.446	217	222	−0.413	0.117
OP178939.1	0.5114	0.2102	0.35	0.1699	0.27	0.082	0.469	47.77	0.301	0.439	209	214	−0.432	0.126
OP178940.1	0.5114	0.2102	0.35	0.1699	0.27	0.082	0.469	47.77	0.301	0.439	209	214	−0.432	0.126
OP178941.1	0.5114	0.2102	0.35	0.1699	0.27	0.082	0.469	47.77	0.301	0.439	209	214	−0.432	0.126
ON864402.1	0.5027	0.2131	0.3512	0.1688	0.274	0.097	0.479	49.57	0.304	0.446	217	222	−0.413	0.117
OP178943.1	0.5198	0.2034	0.3396	0.1765	0.261	0.06	0.455	49.29	0.301	0.441	209	214	−0.385	0.126
OP178944.1	0.5114	0.2102	0.35	0.1699	0.27	0.082	0.469	47.77	0.301	0.439	209	214	−0.432	0.126
OP178945.1	0.5114	0.2102	0.35	0.1699	0.27	0.082	0.469	47.77	0.301	0.439	209	214	−0.432	0.126
ON864403.1	0.5027	0.2131	0.3512	0.1688	0.274	0.097	0.479	49.57	0.304	0.446	217	222	−0.413	0.117
OP178947.1	0.5114	0.2102	0.35	0.1699	0.27	0.082	0.469	47.77	0.301	0.439	209	214	−0.432	0.126
ON864404.1	0.4916	0.2123	0.3136	0.225	0.27	0.113	0.484	53.26	0.344	0.447	215	219	−0.389	0.110
OP178949.1	0.5057	0.2216	0.3522	0.1656	0.269	0.062	0.459	49.65	0.306	0.436	209	214	−0.446	0.126
ON864405.1	0.4918	0.224	0.3593	0.1646	0.267	0.07	0.465	51.77	0.309	0.443	217	222	−0.426	0.117
OP178951.1	0.5114	0.2102	0.35	0.1699	0.27	0.082	0.469	47.77	0.301	0.439	209	214	−0.432	0.126
OP178952.1	0.5114	0.2102	0.35	0.1699	0.27	0.082	0.469	47.77	0.301	0.439	209	214	−0.432	0.126
OP178953.1	0.5114	0.2102	0.35	0.1699	0.27	0.082	0.469	47.77	0.301	0.439	209	214	−0.432	0.126
ON864406.1	0.4972	0.2155	0.3533	0.1698	0.272	0.097	0.479	49.88	0.307	0.448	215	220	−0.407	0.114
OP178955.1	0.5114	0.2102	0.35	0.1699	0.27	0.082	0.469	47.77	0.301	0.439	209	214	−0.432	0.126
OP178956.1	0.5114	0.2102	0.35	0.1699	0.27	0.082	0.469	47.77	0.301	0.439	209	214	−0.432	0.126

CAI - Codon Adaptation Index, CBI - Codon Bias Index, **Aromo** - Aromaticity, **FoP** - frequency of optimal codons, **GRAVY** - The general average hydropathicity or the grand average of hydropathicity.

## 4 Experimental Design, Materials, and Methods

### 4.1 Sample collection

Leaf samples of sixty-two (62) *P. biglobosa* landraces were collected across three agro-ecological areas AEAs (Rainforest, Guinea savannah and Sudan savannah) in Nigeria, following the procedures described by Omonhinmin et al. [3]. The samples were Silica gel-dried, cleaned, assigned accession numbers, and stored in a −80 °C cooling facility at the Molecular Biology Research Laboratory, Department of Biological Sciences, Covenant University, Ota, Nigeria.

### 4.2 Genomic DNA extraction

Genomic DNA was extracted using a modified CTAB-based method [4]. and quality and quantity were authenticated using the ThermoFischer® Nanodrop spectrophotometer ND-8000-GL

### 4.3 PCR amplification and purification

The PCR reactions for the rbcL regions of the 62 samples followed the procedures described by Popoola et al. [7.8]. The final volume of 25 µL consisted of 2 µL of genomic DNA (10–30 ng/µl), 12.5 µL of NEB OneTaq 2X Master Mix with standard buffer, 0.5 µL of rbcL 1F (5-ATGTCACCACAAACAGAAAC-3) and 10 µM of 724R (5GTAAAATCAAGTCCACCGCG-3), [5] and 9.5 µL of nuclease-free water. The PCR reaction conditions and the integrity of the PCR samples followed procedures as described by [[6], [7]]. The PCR products were thereafter cleaned using an enzymatic method (ExoSAP).

### 4.4 Sequencing and data analysis

The PCR amplicons were sequenced at Inqaba biotechnical Industries (Pty) Ltd, South Africa. Sequences were cleaned and aligned using default settings in BioEdit Sequence Alignment Editor [[8], [9]]. Nucleotide sequence statistics of the 62 *P. biglobosa* accessions were performed using MEGAX following the procedure of Tajima et al. [10]. DnaSP v6.12.03 software was employed to determine genetic diversity parameters, multidomain analysis and population structure parameters. The number of sequences per population, the total number of polymorphic sites, the total number of mutations, the average number of nucleotide differences between the sequences, mutation rate per population/sequences, nucleotide diversity, haplotype number and diversity, haplotype diversity variance, gene flow and genetic differentiation among the different populations were estimated. Amino acid residues and codon usage parameters were assessed using CodonW [[10], [11], [12]].

## CRedit Author Statement

**Conrad A. Omonhinmin**: Conceptualization, experiment planning, Reviewing, and Editing. **Nchedo S. Taiwo, Paul B. Okonkwor, Israel M. Ajayi**: Carried out the experiments. **Oluwadurotimi Aworunse**: Writing-Reviewing and Editing. **Joshua Oyekanmi**: Software, Data curation. **Olanrewaju Olufowobi:** Data curation**. Paul A. Akinduti, Ibukun Ajiboye, Olugbenga S. Taiwo, Bosede Temitope, Olubukola Oziegbe, and Adetutu Bello, Margaret Oniha, Oyewumi Oshamika, Eze F. Ahuekwe, Samuel A. Ejoh, Olayemi Akinnola**: Visualization, Investigation. **Solomon Oranusi**: Supervision. **Jacob O. Popoola:** Conceptualization, Data curation, Writing original draft preparation.

## Data Availability

Parkia biglobosa ribulose-1,5-bisphosphate carboxylase/oxygenase large subunit (rbcL) gene, partial cds; chloroplast. (Original data) (GenBank, NCBI).
